# The Box–Behnken Design for Optimizing HPLC Separation and Validation of Astilbin in *Lysiphyllum strychnifolium* Stems

**DOI:** 10.1155/sci5/6177990

**Published:** 2024-12-24

**Authors:** Chaowalit Monton, Jirapornchai Suksaeree

**Affiliations:** ^1^Drug and Herbal Product Research and Development Center, College of Pharmacy, Rangsit University, Muang, Pathum Thani 12000, Thailand; ^2^Department of Pharmacognosy, College of Pharmacy, Rangsit University, Muang, Pathum Thani 12000, Thailand; ^3^Department of Pharmaceutical Chemistry, College of Pharmacy, Rangsit University, Muang, Pathum Thani 12000, Thailand

**Keywords:** astilbin, box–behnken design, chemical marker, HPLC separation, *Lysiphyllum strychnifolium*

## Abstract

The goal of the research was to use BBD, a productive RSM approach, to enhance the HPLC separation and validation of astilbin in *Lysiphyllum strychnifolium* stems. The percentage of acetonitrile (ACN), flow rate, and temperature were among the independent parameters that determined how much the chromatographic condition chosen from factor-level screens lowered the *t*_*R*_ of astilbin. The six dependent variables were *t*_*R*_, PA, *k*′, Rs, N, and As. The following HPLC settings were optimal for astilbin separation: 19% ACN at *t*_0_–*t*_15_, 0.8 mL/min flow rate, and 25°C temperature, resulting in a 26-min reduction in working time. This resulted in a separation success rate of 68.57%. Findings revealed the following sequence for *t*_*R*_, PA, *k*′, Rs, *N*, and As: 12.108 ± 0.010 min, 78,845,108 ± 420,267, 2.510 ± 0.003, 2.141 ± 0.024, 10,945 ± 80, and 0.991 ± 0.005. The limit of detection was 0.10 μg/mL, while the limit of quantitation was 0.20 μg/mL. The calibration curve was constructed using concentrations ranging from 0.39 to 50 μg/mL, with an *R*^2^ value of 0.9991, indicating excellent linearity. The intraday and interday precision RSD values were 0.069%–1.892% and 0.993%–3.229%, respectively. Recovery values were between 95.56% and 105.57%, confirming the method's accuracy. Astilbin was found at 175.51 ± 7.80 μg in *L. strychnifolium* stem extracts; its actual concentration was 3.51 ± 0.16%. The usefulness of astilbin as a chemical marker in *L. strychnifolium* stems may therefore be determined based on the criteria that have been established using this information.

## 1. Introduction

**Figure 1:**
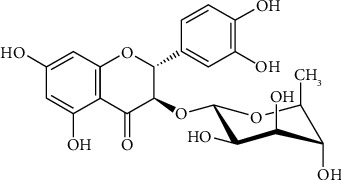
Chemical structure of astilbin.

Flavonoids are secondary metabolites produced by plants. Natural flavonoids include the phenolic structures. It is possible to discover them in a variety of plant parts, including fruits, rhizomes, flowers, leaves, and other parts [1, 2]. Flavonoids offer several health benefits. Flavonoids exhibit antioxidant characteristics, immunomodulators, anticancer agents, antiviral agents, antibacterial agents, anti-inflammatory agents, and many other health benefits [3–5]. Flavonoids are valuable to cosmetics, pharmaceuticals, nutraceuticals, and other industries due to their various benefits [6, 7]. Recently, flavonoids have been intensively explored. Many studies have extracted, identified, and studied its characteristics in vitro and in vivo [8]. Astilbin is one of these flavonoid compounds. Astilbin is a dihydroflavonol rhamnoside found in various plants such as *Engelhardtia chrysolepis* [9], *Vitis vinifera* [10], *Smilax aristolochiifolia* [11], *Smilax glabra* [12], and *Lysiphyllum strychnifolium* (*L. strychnifolium*) [13]. Astilbin is known by its IUPAC designation, (2R,3R)-2-(3,4-dihydroxyphenyl)-5,7-dihydroxy-3-[(2S,3R,4R,5R,6S)-3,4,5-trihydroxy-6-methyloxan-2-yl]oxy-2,3-dihydrochromen-4-one, and typically, it is (2R,3R)-taxifolin-3-O-R-L-rhamnopyranoside [14], displayed in Figure 1. Plants and herbs containing astilbin were applied historically in China and some other Asian nations in the care of different ailments including hyperuricemia, chronic renal failure, and immunosuppressant. [15, 16].

*L. strychnifolium* (Craib) A. Schmitz (synonym *Bauhinia strychnifolia* Craib.) is a woody climbing tree that has the Thai common name Ya Nang Daeng. It is a local plant found only in Thailand and is a member of the Fabaceae family. Furthermore, it is found mostly in northern Thailand's dry deciduous dipterocarp forests. There is a variance in the morphological traits of the foliage in an intraspecific population in addition to the species [17]. This plant has traditionally been utilized in Thailand to avoid pesticide poisoning, as well as to treat fever and alcoholism. The plant's traditional applications for detoxification and chronic disease prevention are supported by the antioxidant activity obtained from extracts of the leaves and stems [18]. Furthermore, HPLC comparative research suggested that gallic acid, quercetin, and trilobatin from *L. strychnifolium* leaves are the major components that can be used as chemical markers for quality assessment [19–21], as well as astilbin from its stem [13, 22]. All earlier HPLC comparisons indicate that analyzing astilbin from extracts takes more than 60 min. Therefore, while producing plant extracts as pharmaceutical products, it is a waste of time to research the important substances in them [13, 22]. Therefore, rapid analysis time is essential to make it simple and fast for assessing substances. The development of an optimal analytical technique requires a thorough assessment of the factors affecting chromatographic performance. Finding the best operating conditions and analyzing the relationship between the parameter settings and the resulting performance are two frequent purposes for the design of experiment techniques, and a statistical model may be applied.

A potent technique that may be used to assess several factors with a minimal number of experimental trials is response surface methodology (RSM) [23]. An optimum response may be found by analyzing the impact of various factors on the response variable via the use of an experimental design developed through a combination of statistical and mathematical processes. Because Box–Behnken design (BBD) is an extremely practical and effective RSM approach, it was used in this study to optimize process variables. In addition to having extremely strong symmetry and rotatability, these designs provide fewer experimental runs in comparison with the widely used central composite design and provide maximum information. Three levels (−1, 0, 1) are needed for BBD as a response surface design, and it may be applied to several parameters ranging from 3 to 21. Both numerical and categorical variables may be used for optimization in a BBD; however, using categorical factors often results in more runs [24–26]. These may be effectively used for a range of process optimization of HPLC separation because of the high resolution of BBD. Effective chromatographic analysis requires the simultaneous optimization of many dependent variables, which is where BBD comes in helpful. For levofloxacin and moxifloxacin analysis, the most substantial variables that affected the results are the retention time, relative retention time, peak symmetry, tailing factor, number of theoretical plates, Foley–Dorsey parameter, resolution factor, and peak width at half height. The response under the given circumstances may be predicted due to the polynomial equations that characterize the mutual interactions between the independent variables. The observed and calculated values of the dependent variables using the polynomial equations are comparable. It verified that the applied design was appropriate [27]. Similarly, the reports have also been made on the usage of BBD to provide optimal conditions for the analysis of (i) methocarbamol, indomethacin, and betamethasone [28], (ii) febuxostat [29], (iii) rivastigmine hydrogen tartrate and asiaticoside [30], (iv) paracetamol and diclofenac sodium [31], (v) hydrochlorothiazide [32], (vi) curcumin [33], (vii) paclitaxel [34], and (viii) thymoquinone [35]. Nevertheless, there have been no previous reports of using BBD to give optimal conditions for astilbin analysis.

Consequently, since BBD is a very practical and effective RSM approach, it was used in this study to optimize process variables. It can identify the components of the model, identify instances where the model does not fit, and seem more appealing when the points are located in the middle and at the midpoints of the process's edges. Therefore, the primary goal of the current study was to improve the HPLC separation procedure to evaluate the impact of the percentage of acetonitrile (ACN; *A*), flow rate (*B*), and temperature (*C*) process conditions. A series of tests were conducted using values of *A*, *B*, and *C* within the range that was appropriate. Astilbin analysis would benefit from a thorough understanding of the combined effects of process parameters and additives on the HPLC separation method. Investigations were performed on the effects of percentage of ACN, flow rate, and temperature individually as well as the combinations of all three factors. The findings of this research will help determine the ideal parameters for the HPLC separation process of astilbin in *L. strychnifolium* that operates under different situations. The outcomes of the characterization and optimization may also be utilized as guidelines for evaluating the chemical marker's quality.

## 2. Materials and Methods

### 2.1. Materials

The *L. strychnifolium* stems came from a Rangsit market, Pathum Thani, Thailand, and originated from a farm in Phetchabun, Thailand. Nirun Vipunngeun identified the *L. strychnifolium* stems. A voucher specimen of *L. strychnifolium* stems was stored at the Drug and Herbal Product Research and Development Center, College of Pharmacy, Rangsit University. It was given the number JS-LS1-06-21. Astilbin standard had been purchased from Sigma-Aldrich, USA. We got high-quality laboratory-grade chemicals from Fluka (USA), Sigma-Aldrich (USA), and Merck (Germany).

### 2.2. Preparation of *L. strychnifolium* Stem Extracts

An *L. strychnifolium* stem sample that had been dried was crushed, pulverized, and then passed through a 40-mesh sieve to produce a powder. Sieves are commonly used in quality control and pharmaceutical production to measure the sample particle size. The most used measuring unit for sieves and screens is mesh. The *L. strychnifolium* stem powder had a particle size of 420 μm after passing through a 40-mesh sieve. A 1000-mL beaker was filled with 200 mL of the 80% ethanol solvent after 5 g of *L. strychnifolium* stem powder was weighed in it. A microwave oven (model MS23F300EEK, Samsung Electronics Co., Ltd., Malaysia) was used to do the extraction. For the appropriate extraction period, intermittent microwave radiation was delivered (the oven was switched “on”); to prevent overheating the extraction solvent, the oven was turned “off.” One extraction cycle, 20 s of extraction time, and 800 W of power were required for microwave-assisted extraction. After extraction, the liquid phase was separated and filtered using a 0.45-μm Whatman No. 1 filter paper by suction filtration with a waiting time of less than 1 min. The liquid was then concentrated using a rotary evaporator at 40°C–60°C under vacuum. The research employed *L. strychnifolium* extracts with a yield of 12.05 ± 0.14%.

### 2.3. Screening Factor Levels by One-Factor-at-a-Time (OFAT) and Optimization of Astilbin Separation in *L. strychnifolium* Stem Extracts by BBD

The *L. strychnifolium* stem extracts were prepared in this approach, and a concentration of 1 mg/mL was used for the screening factor levels and optimization. The first step was to screen the factors of HPLC conditions for astilbin separation using the traditional OFAT approach. This method allowed for the modification of just OFAT, while the other variables were held constant.

Then, several variables could be changed at the precise same time. This was essential for determining how two or more factors influenced responses in a short number of experimental runs, and it required just a small amount of resources in terms of both time and materials to collect the whole quantity of data that were required. The BBD optimization of the proposed method involved three independent and six dependent variables. BBD produces higher-order response surfaces while requiring fewer experiments than other factorial techniques [36]. This design considered *A*, *B*, and *C* as independent factors, and the retention time (*Y*_1_), peak area (*Y*_2_), capacity factor (*Y*_3_), resolution (*Y*_4_), plate number (*Y*_5_), and asymmetry factor (*Y*_6_) as dependent variables. A preliminary analysis was done to identify the maximum and lower limits of these essential characteristics. We selected the following range of independent factors for this experiment: the percentage of ACN varied from 15% to 20%, the flow rate varied from 0.8 to 1.2 mL/min, and the temperature ranged from 25°C to 35°C (Table 1). The statistical analysis was evaluated, and Design Expert software (Version 11, Stat-Ease Inc., Minneapolis, MN, USA) was used for generating experimental designs.

### 2.4. Creation of Design Space and Selection of an Optimal Condition

The design space was established based on the goals for all responses shown in Table 1: retention time (*t*_*R*_) < 15 min, capacity factor (*k*′) > 2, resolution (Rs) > 2, plate number (*N*) > 2000, and asymmetry factor (As) > 0.95. The optimal condition was selected from those that fell within the design space. This condition was then used to reanalyze *L. strychnifolium* stem extracts to confirm the accuracy of the predictions made by the computer program, Design Expert software.

### 2.5. HPLC Instrument

The study was carried out using high-performance liquid chromatography (HPLC) equipment, namely, a 1260 Infinity system from Agilent, USA. The separation column used was a Luna 5u C18(2) 100A separation column, measuring 150 × 4.6 mm, with an internal diameter of 5 μm. It was a gradient elution system that had a flow rate of 0.8–1.2 mL per minute and a column temperature of 25°C–35°C, based on the experimental design as earlier mentioned. ACN and 2% v/v acetic acid were the components that made up the mobile phase. A wavelength of 290 nm was chosen for UV detection. The volume of the injection was 10 μL.

### 2.6. HPLC System Suitability

The study examined four distinct parameters: resolution, asymmetry, capacity factor, and number of theoretical plates. All of these data were collected using the HPLC program. *L. strychnifolium* stem extracts at 1 mg/mL in 80% ethanol, and astilbin standard at 25 μg/mL in methanol were both used in 10 repeats of this determination. Acceptable values were established to be more than 2000 theoretical plates, more than 2 for the capacity factor, close to 1 for the asymmetry, and more than 2 for the resolution [37].

### 2.7. HPLC Method Validation

The ICH guideline was followed for doing method validation. This was done for five different topics: linearity and range, limit of detection and limit of quantitation, specificity, precision, and accuracy. The stock solution of astilbin standard was made in 500 μg/mL in methanol that was used to dilute in varied concentrations for the next step given below.

#### 2.7.1. Linearity and Range

This stock solution was further diluted to reach concentrations of 50, 25, 12.5, 6.25, 3.125, 1.5625, 0.78125, and 0.390625 μg/mL. Filtration was performed on these diluted solutions using a 0.45-μm-pore nylon syringe filter. They were then injected into the HPLC instrument. Three analyses of each concentration were performed. Three important features were determined in the process of generating a calibration curve for astilbin: the linear equation, the coefficient of determination (*R*^2^), and the testing range.

#### 2.7.2. Limit of Detection and Limit of Quantitation

Astilbin was diluted and then injected into an HPLC apparatus for analysis to examine the limit of detection and the limit of quantitation. The limit of quantitation for astilbin was the concentration at which the signal-to-noise ratio was 10:1. Additional dilutions were then tested using the HPLC apparatus, and the concentration at which astilbin yielded a signal-to-noise ratio of 3:1 was declared as the limit of detection.

#### 2.7.3. Specificity

UV spectra of the astilbin peak, which may be observed in stem extracts of *L. strychnifolium*, were obtained by utilizing an HPLC apparatus. If the UV spectra from this segment were similar to the UV spectrum of the recognized astilbin standard and matched each other, the specificity was considered sufficient understanding.

#### 2.7.4. Precision

The astilbin standard was prepared, filtered, and put into HPLC analysis (*n* = 3) at three different concentrations: 3.125 μg/mL, 12.5 μg/mL, and 25 μg/mL. Intraday precision was defined as the percentage relative standard deviation (% RSD) obtained from the analyses performed during the same day. Interday precision was measured as the % RSD from analyses performed over three consecutive days.

#### 2.7.5. Accuracy

A spiking approach was used to evaluate the accuracy. A 1 mg per milliliter ethanol extract of the stem of *L. strychnifolium* was made using 80% ethanol. One milliliter of this extract was loaded into a five-mL volumetric flask. The volumetric flask was then filled with an astilbin standard solution mixed with the *L. strychnifolium* stem extract. Astilbin was present in the combination at concentrations of 3.125 μg/mL, 12.5 μg/mL, and 25 μg/mL. These combinations were then filtered using a syringe filter with a pore size of 0.45 μm, and then they were examined using an HPLC apparatus (*n* = 3). Recovery percentages were recorded for each concentration level.

#### 2.7.6. Robustness and Ruggedness

The robustness of the optimized HPLC method was evaluated by introducing small variations in key parameters such as mobile phase composition (ACN percentage), flow rate (±0.1 mL/min), and column temperature (±5°C). The method was considered robust if there were no significant changes in chromatographic performance, including retention time, resolution, and peak area. Ruggedness was tested by performing the method with different operators and instruments, and on different days. The RSD was calculated for each parameter, and the method was deemed rugged if the results showed minimal variation (RSD < 5%) across these conditions, ensuring consistent performance in various laboratory settings.

## 3. Results and Discussion

### 3.1. Medium Optimization Through OFAT

**Figure 2:**
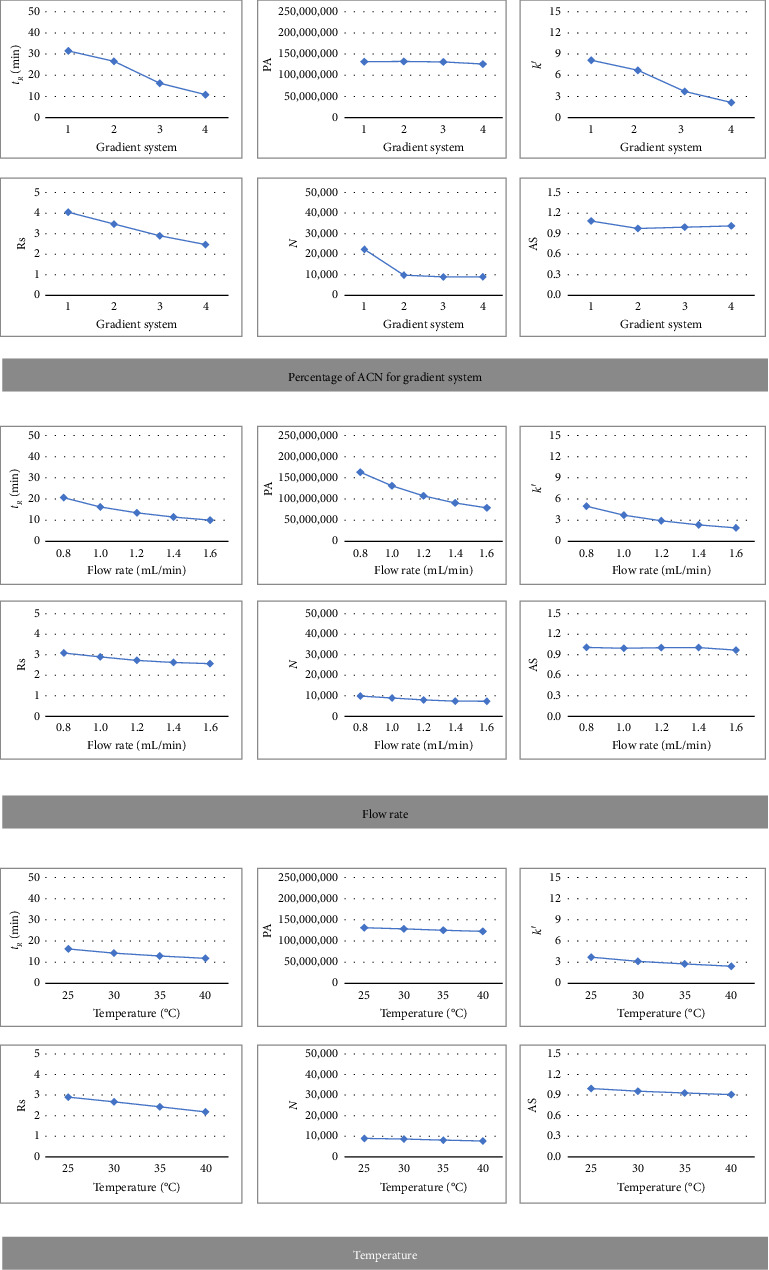
One-factor-at-a-time analysis of percentage of ACN, flow rate, and temperature on astilbin separation.

Before large-scale manufacturing, optimizing independent variables is necessary to maximize a given product's output. It is possible to use a variety of optimization techniques, from the traditional OFAT method to more modern statistical and mathematical techniques. There are both advantages and disadvantages to every method. A desired outcome may be obtained by combining several optimization techniques [38]. The variables were assessed via the use of OFAT to determine the best conditions for the factorial design. All other experimental conditions were held constant while the optimal value of each of the chosen variables was assessed. The initial factor-level screening of A, B, and C is shown in Figure 2.  Table 2 displays the screenings of the percentage of ACN in gradient elution systems, flow rate, and temperature for the HPLC method.

The influence of the ACN percentage in the mobile phase on several chromatographic parameters during the HPLC analysis of astilbin was extensively explored. Because of the lower polarity of the mobile phase, which resulted in weaker interactions between the analyte and the stationary phase and accelerated the elution of astilbin, *t*_*R*_ dropped as the amount of ACN increased. This decrease in the retention time was following typical results seen at greater concentrations of organic solvents, including ACN. The PA initially increased with increasing ACN percentages, possibly because of improved solubility and better peak shape. However, at high concentrations, the peak area began to decrease, probably due to poor interaction and insufficient analyte retention. As the concentration of ACN increased, *k*′ decreased indicating that as the mobile phase became less polar, the retention of astilbin was reduced. Because the decreased retention resulted in larger peaks and a reduction in the differential retention of closely eluting chemicals, Rs exhibited a similar decreasing trend at higher ACN percentages, affecting separation efficiency. Higher ACN concentrations also caused a decrease in *N*, which could be an indication of decreased column efficiency and wider peak widths caused by less successful analyte–stationary phase interactions. As increased with higher ACN percentages, indicating peak tailing and asymmetry. This was probably caused by several objects, such as changed mass transfer properties in the mobile phase and lower retention. These results demonstrated the necessity of carefully adjusting the mobile phase composition for balanced chromatographic performance in the analysis of astilbin, as increasing the ACN percentage enhanced peak solubility and decreased the retention time but adversely affected resolution, column efficiency, and peak symmetry.

Several important associations were found when the impact of flow rate on chromatographic parameters during the HPLC analysis of astilbin was methodically investigated. The expected negative correlation between the flow rate and *t*_*R*_ was observed, as *t*_*R*_ decreased as the flow rate increased. This was because analytes worked less time in the stationary phase. Concurrently, with the modest increases in flow rate, the PA first increased; but, at higher flow rates, it started to decrease, most likely because of insufficient analyte–stationary phase interactions, which resulted in incomplete peak formation. Additionally, as the flow rate increased, *k*′ dropped, indicating lower retention of astilbin in the column. Higher flow rates had a negative influence on Rs, making it difficult to separate closely eluting peaks because shorter equilibration times produced larger peaks. As the flow rate increased, N decreased, suggesting reduced column efficiency and wider peaks due to less time for analytes to equilibrate between mobile and stationary phases. Increased flow rates led to a greater As, indicating a decrease in peak symmetry due to insufficient mass transfer and reduced stationary phase interaction. These results showed that whereas faster flow rates shortened analysis times, they also affected peak quality, resolution, and efficiency. This emphasizes the need for flow rate optimization to achieve a balance between chromatographic performance and analysis efficiency.

The impact of temperature on a variety of chromatographic characteristics was examined during the HPLC analysis of astilbin. These parameters included *t*_*R*_, PA, *k*′, Rs, N, and As. *t*_*R*_ often reduced as temperatures increased, probably because of the mobile phase's lower viscosity, which increased analyte diffusion. A lower interaction between astilbin and the stationary phase was shown by a decline in *k*′ that corresponded with this reduction in *t*_*R*_. Higher temperatures usually result in a steady PA, while some variations could occur because of variations in the analyte's solubility or the detector's response. Because of the reduced selectivity between the solute and stationary phase, Rs between closely eluting peaks was negatively impacted at higher temperatures, resulting in wider peaks. A decrease in the As suggested a loss of peak symmetry at higher temperatures, while N tended to decrease with temperature, suggesting a decrease in column efficiency, possibly as a result of the solute's decreased interaction with the stationary phase. The significance of balancing the opposing impacts on chromatographic performance during the HPLC analysis of astilbin was highlighted by these results.

In summary, it was found that *t*_*R*_, *k*′, Rs, and N had significantly decreased. Although they were found to be decreasing, they stayed at acceptable standards in their normal levels [37]. Furthermore, all decreasing responses were affected by temperature and flow rate. The strategy of OFAT was easy to understand. However, for optimizing these reaction parameters using the traditional OFAT approach, a significant number of observations and tests would be necessary.

It was costly and time-consuming and did not investigate how different variables interacted, particularly when carrying out more studies. This was because only one of the variables could be varied at a time, while the other variables had a fixed value. Thus, this was not only time-demanding but also not economically practical. Statistical and mathematical methods, on the other hand, are efficient and have the potential to circumvent these limits altogether. A multifactor approach is necessary for these techniques. The OFAT strategy used to screen interactions between multiple variables and describe the function of the interactions of each component in the process is not as successful as these other approaches. The most efficient of these techniques is RSM, which is a good design model that studies several parameters influencing responses by modifying them in a small number of tests and explains the total effect [39, 40]. Consequently, the chromatographic condition that was composed of several independent variables, including A, B, and C, was selected from the factor-level screenings. This was because these variables demonstrated the capability to significantly reduce the *t*_*R*_ of astilbin in comparison with the initial chromatographic condition. There is also the potential that the data might be used as criteria for assessing the quality of the astilbin as a chemical marker in *L. strychnifolium*. This is another option that is included in the goal.

### 3.2. BBD for Optimization of Significant Variables

**Figure 3:**
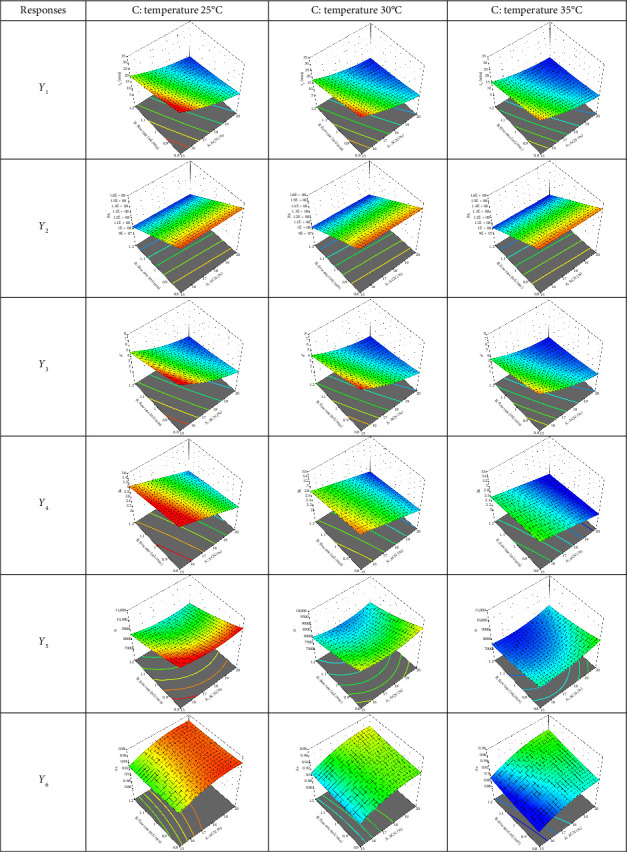
Response surfaces of dependent variables of astilbin separation based on BBD.

The formulation was further optimized using the BBD approach to produce higher-order response surfaces requiring fewer studies. The percentage of ACN, flow rate, and temperature were the three independent variables that were taken into consideration. The six dependent variables were retention time, peak area, capacity factor, resolution, plate number, and asymmetry factor shown to be significantly influenced by each of the independent variables, according to the analysis of variance (ANOVA) (Table 3). In Figure 3, the effects of the investigated independent variables on the dependent responses were shown by the generation of three-dimensional response surface plots derived from the coded equations.

Statisticians utilize the coefficient of determination, or *R*^2^, to evaluate how well the expected regression model matches the study data points. An *R*^2^ score that is closer to 1 is regarded as significant since it indicates that the model equation will fit the research data points well [41]. All the responses (*Y*_1_–*Y*_6_) had an *R*^2^ of more than 0.90, indicating that the input variable was significant in this investigation. However, whether the input variable was important, the *R*^2^ value was always increased when it was added to the model formulation. Consequently, the usual coefficient of determination is replaced with the adjusted *R*^2^. The primary reason for this is that the addition of an input variable does not always increase the adjusted *R*^2^. The adjusted *R*^2^ value is essentially decreased by including unnecessary input variables. Predicted *R*^2^, or the predicted coefficient of determination for the forecast model, is another important concept [42]. Every model had a difference between adjusted *R*^2^ and predicted *R*^2^ of less than 0.2, and all of them had scores over 0.7, indicating that they were dependable and appropriate models. Except for Response *Y*_6_, the adjusted *R*^2^ of 0.9207 was not precisely comparable to the predicted *R*^2^ of 0.5453 that we would expect, with a difference of more than 0.2. This suggested that either a significant block effect occurred or there was an issue with the data or the selected model type. Analyzing the response transformation, outliers, and model reduction parameters was beneficial.

#### 3.2.1. *Y*_1_: Retention Time (Min; *t*_*R*_)

The three independent variables were used to improve the retention time. The quadratic model's ANOVA revealed an excellent BBD surface response. The statistical measure known as the *F*-value, which could be seen in the ANOVA, is used to assess the degree of significance of the means between variables. An *F*-test indicates to the reader if a group of input variables is mutually significant in statistics, as opposed to the *t*-test, which indicates to the reader whether one input variable is extremely significant [43]. The *F*-value of 467.29 indicated that the built-in model was significant. The probability of an *F*-value this big occurring due to noise was < 0.0001. The significance of the model terms might be attributed to *p* values below 0.0500. The essential model parameters in this instance were represented by the equation below, where “*A*” represents the percentage of ACN, “*B*” represents the flow rate, and “*C*” represents the temperature. *A*, *B*, *C*, AB, AC, BC, *A*^2^, *B*^2^, and *C*^2^ were the variables used in the ANOVA for the quadratic model. The coefficients AB, AC, *A*^2^, and *B*^2^ in the present case had *p* values less than 0.05, indicating that adding these coefficients would enhance the model fit. A signal-to-noise ratio of more than four is preferred [44], and it is measured with adequate precision. In this case, the signal level of 75.85 was appropriate. The model could predict results within its specified area; additional testing was not required.

(1) $$\begin{array}{c} Y_{1}=299.195-18.724\;\left(A\right)-107.048\;\left(B\right)-2.128\;\left(C\right)+3.084\;\left(A\right)\left(B\right)+0.078\;\left(A\right)\left(C\right)+0.297\;\left(B\right)\left(C\right)+0.305\;\left(A^{2}\right)+13.945\;\left(B^{2}\right)+0.003\;\left(C^{2}\right). \end{array}$$

#### 3.2.2. *Y*_2_: Peak Area (PA)

Excellent BBD surface response was demonstrated by *A*, *B*, and C's ANOVA for the linear model. The following equation describes the essential model parameters in this case. The built-in model was shown to be significant by the *F*-value of 125.95. There was less than a 0.0001 chance that noise would cause an *F*-value this large. The importance of the model terms might be assigned to *p* values < 0.0500. Unfortunately, only coefficient B's *p* value was less than 0.05, suggesting that including these coefficients might improve the model's fit. The signal strength of 30.46 was suitable. There was no need for further testing since the model could forecast outcomes within its defined range,

(2) $$\begin{array}{c} Y_{2}=271123163.025-795307.967\;\left(A\right)-126382234.167\;\left(B\right)-110731.517\;\left(C\right). \end{array}$$

#### 3.2.3. *Y*_3_: Capacity Factor (*k*′)

The ANOVA for the quadratic model by *A*, *B*, *C*, AB, AC, BC, *A*^2^, *B*^2^, and *C*^2^ showed an excellent BBD surface response. The important model parameters in this instance were represented by the equation that follows. The F-value of 467.23 demonstrated the significance of the built-in model. The probability of noise producing a significant *F*-value was less than 0.0001. Model terms with *p* values < 0.0500 might be considered significant. Coefficients AB, AC, *A*^2^, and *B*^2^ had *p* values less than 0.05, suggesting that adding them might improve the model fit. The signal strength of 75.84 was adequate. Since the model could predict results within its given range, no more testing was required,

(3) $$\begin{array}{c} Y_{3}=85.727-5.428\;\left(A\right)-31.027\;\left(B\right)-0.617\left(C\right)+0.894\;\left(A\right)\left(B\right)+0.023\;\left(A\right)\left(C\right)+0.086\;\left(B\right)\left(C\right)+0.088\;\left(A^{2}\right)+4.039\left(B^{2}\right)+0.001\;\left(C^{2}\right). \end{array}$$

#### 3.2.4. *Y*_4_: Resolution (Rs)

The BBD surface response was very good in the ANOVA for the 2FI model by A, B, C, AB, AC, and BC. The equation that follows represents the significant model parameters in this case. The relevance of the built-in model was proven by the F-value of 182.76. There was less than a 0.0001 chance of noise generating a significant F-value. Model terms that had *p* values less than 0.0500 might be significant. *p* values for the coefficients AB and BC were less than 0.05, indicating that including them might enhance the model's fit. There was no need for further testing since the 47.98 signal strength was acceptable,

(4) $$\begin{array}{c} Y_{4}=12.206-0.375\;\left(A\right)-2.803\;\left(B\right)-0.182\;\left(C\right)+0.062\;\left(A\right)\left(B\right)+0.006\;\left(A\right)\left(C\right)+0.039\;\left(B\right)\left(C\right). \end{array}$$

#### 3.2.5. *Y*_5_: Plate Number (*N*)

The BBD surface response in the ANOVA for the quadratic model by *A*, *B*, *C*, AB, AC, BC, *A*^2^, *B*^2^, and *C*^2^ was excellent. The important model parameters in this instance are represented by the following equation. The *F*-value of 115.42 demonstrated the applicability of the built-in model. The probability of noise producing a significant *F*-value was less than 0.0001. *p* values under 0.0500 indicated possible significance for model terms. *p* values for the factors AC, BC, *A*^2^, and *B*^2^ were less than 0.05, suggesting that including them might improve the model's fit. Since the 38.66 signal strength was satisfactory, no more testing was required,

(5) $$\begin{array}{c} Y_{5}=52051.058-2299.003\;\left(A\right)-17798.750\;\left(B\right)-776.572\;\left(C\right)-20.167\;\left(A\right)\left(B\right)+17.040\;\left(A\right)\left(C\right)+246.250\;\left(B\right)\left(C\right)+51.952\;\left(A^{2}\right)+3836.250\;\left(B^{2}\right)+1.961\;\left(C^{2}\right). \end{array}$$

#### 3.2.6. *Y*_6_: Asymmetry Factor (As)

The appropriateness of the suggested approach was shown by the peak's form and symmetry. We used the BBD surface response and ANOVA to optimize the tailing factor value given the quadratic model. The following equation represents the significant model parameters in this case. The built-in model's applicability was shown by the F-value of 21.64. Less than 0.0001 was the possibility of noise generating a significant F-value. Terms in the model that have *p* values less than 0.0500 could be significant. Variables BC and *A*^2^ had *p* values less than 0.05, suggesting that including them might enhance the model's fit. No more testing was necessary since the 17.25 signal strength was enough,

(6) $$\begin{array}{c} Y_{6}=0.890+0.062\;\left(A\right)-0.382\;\left(B\right)-0.021\;\left(C\right)+0.005\;\left(A\right)\left(B\right)+0.001\;\left(A\right)\left(C\right)+0.007\;\left(B\right)\left(C\right)-0.002\;\left(A^{2}\right)+0.063\;\left(B^{2}\right)+0.001\;\left(C^{2}\right). \end{array}$$

**Figure 4:**
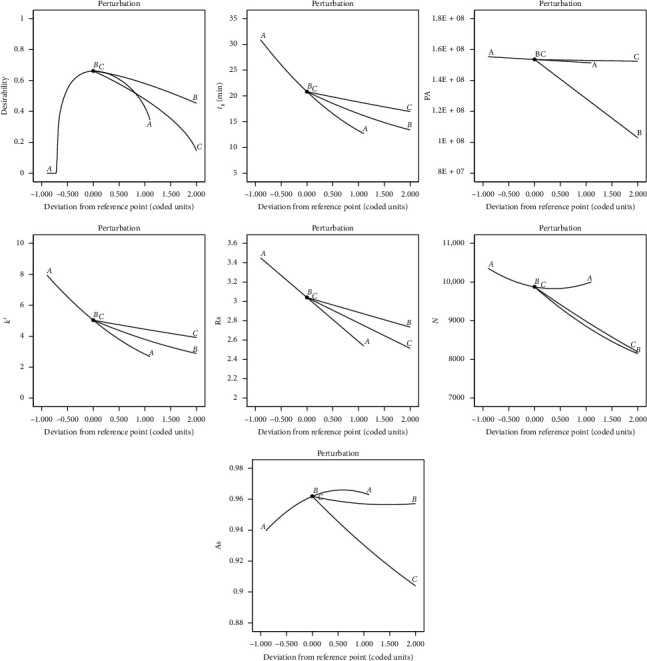
Perturbation plots of all responses (*Y*_1_–*Y*_6_).

The perturbation plot, shown in Figure 4, showed the relative impact of each element at a specific location in the design space. This graphic helps in determining the element that affects every answer the most. All the answers were sensitive to every factor taken into consideration in this investigation, as shown by the considerable curvature of factors *A*, *B*, and *C*. The increased slope of factor *A* suggested that they were highly responsive to *t*_*R*_, *k*′, and Rs. They were very sensitive to the PA and *N*, as seen by the greater slope of factor *B*. The higher slope of factor *C* suggested their strong sensitivity to As.

### 3.3. Optimal Condition

**Figure 5:**
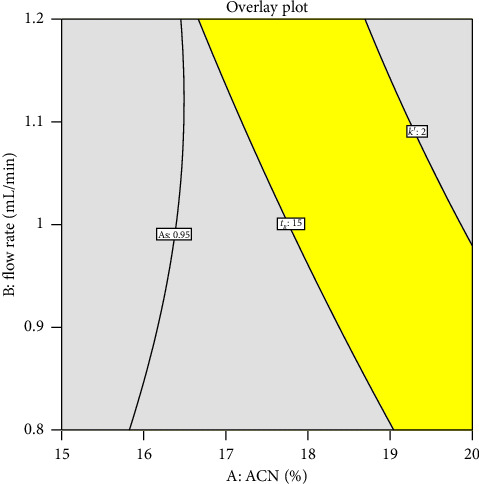
Design space for astilbin separation when temperature of 25°C was used. The yellow area demonstrated that *t*_*R*_ < 15 min, *k*′ > 2, Rs > 2, *N* > 2000, and As > 0.95.

Although all of the factors affected every response, *t*_*R*_ was the primary goal, while several responses had values within the range that met the requirements for an HPLC system that was deemed suitable. Consequently, the criteria for optimizing are described in Table 1.  Table 4 displays the optimum HPLC condition for astilbin separation. The design space revealed a high region when the temperature of astilbin separation was set at 25°C (Figure 5). Therefore, the percentage of ACN at *t*_0_–*t*_30_ was 19%, with a flow rate of 0.8 mL/min at a temperature of 25°C was selected as an optimal condition. The astilbin separation in *L. strychnifolium* stem extracts under optimal HPLC conditions was repeated to confirm the accuracy of the prediction of the model.  Table 5 displays the findings, which showed very low percent errors for all parameters. The remarkable degree of agreement between the experimental and anticipated results highlights the precision and dependability of the computer software's predictions.

**Figure 6:**
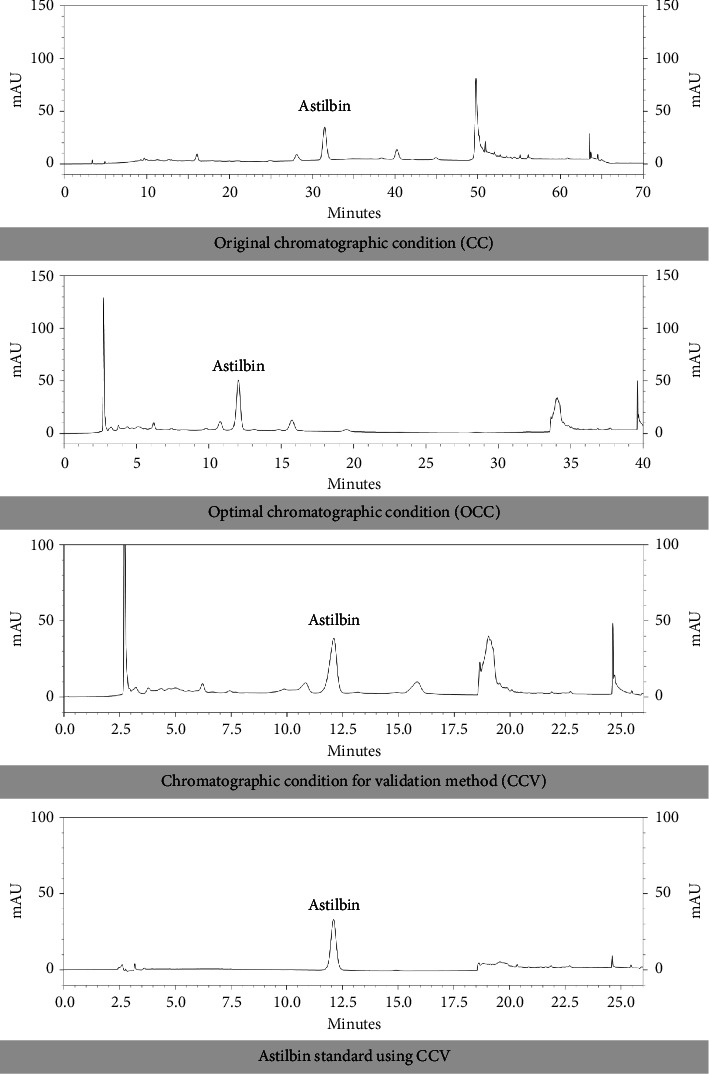
HPLC chromatograms of astilbin separation in *L. strychnifolium* stem extracts (1 mg/mL in 80% ethanol) from different chromatographic conditions (CC, OCC, and CCV systems) and HPLC chromatogram of astilbin standard (25 *μ*g/mL in methanol) using CCV system.

Table 4 and Figure 6 from the CC demonstrate that the separation of astilbin from stem extracts of L. strychnifolium occurred at 70 min, with a retention time (*t*_*R*_ of astilbin recorded at 31.50 min. The duration might be shortened to 40 min under OCC, showing the *t*_*R*_ of astilbin at 12.04 min. Consequently, this effort could reduce the approximate work time by 42.86%, from 70 min to 40 min. It could be observed from Figure 6 that the *t*_*R*_ of astilbin was eluted with just 19% of ACN. Therefore, we developed a new method for further use as a CCV. The overall time worked might have lowered to 26 min, while the *t*_*R*_ of astilbin remained at 12.10 min. Reduction time working for astilbin separation in *L. strychnifolium* stem extracts was successfully developed, with a 68.57% success rate. The astilbin standard was prepared and analyzed using CCV, which confirmed the presence of astilbin in *L. strychnifolium* stem extracts. This condition might serve as a set of recommendations for assessing the quality of the chemical marker.

### 3.4. HPLC Method Validation

In the beginning, we concluded that a gradient elution system consisting of ACN and 2% v/v acetic acid at 25°C of temperature separation was necessary for the validation of the HPLC technique utilized to perform the separation and identification of astilbin in *L. strychnifolium* stem extracts. This is presented in Table 4 with a CCV elution of 26 min. A suitability test for the CCV gradient elution system was verified and evaluated. The findings showed that the following was the sequence of *t*_*R*_, PA, k', Rs, N, and As: 12.108 ± 0.010 min, 78,845,108 ± 420,267, 2.510 ± 0.003, 2.141 ± 0.024, 10,945 ± 80, and 0.991 ± 0.005. These results were all within acceptable ranges [37].

The limit of detection was 0.10 μg/mL, while the limit of quantitation was 0.20 μg/mL. The calibration curve was determined using a diluted stock solution of astilbin standard, ranging from 0.39 to 50 μg/mL. The findings indicated an accomplishment of great linearity. The linear equation for standard astilbin was *y* = 3,044,115.78x + 1,003,379.15 followed by an *R*^2^ value of 0.9991.

**Figure 7:**
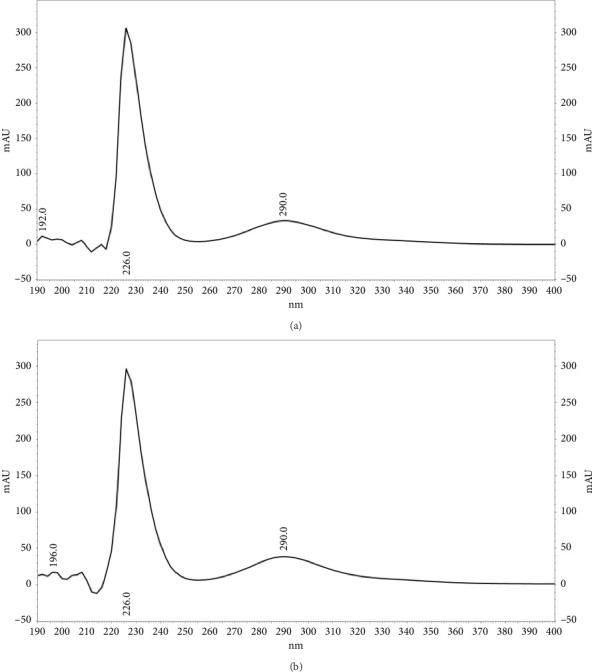
UV spectra of (a) astilbin standard (25 μg/mL in 80% methanol) and (b) astilbin in *L. strychnifolium* stem extracts (1 mg/mL in 80% ethanol) using CCV system.

The UV spectra of the astilbin discovered in the stem extracts of *L. strychnifolium* correlated with those of the astilbin standard (Figure 7). The specificity of the analytical procedure was highlighted by this similarity.

The % RSD values for intraday and interday precision varied from 0.069% to 1.892% and 0.993%–3.229%, respectively. These low RSD values were indicative of the great degree of precision offered by this approach. The procedure displayed accuracy as evidenced by the recovery values close to 100%, particularly in the range of 95.56 ± 2.13% to 105.57 ± 3.21%. The accuracy of the approach was tested using recovery assessments.

The results of the robustness and ruggedness testing demonstrated that the optimized HPLC method was both robust and rugged. The method exhibited a minimal variation in chromatographic performance when small deliberate changes were made to key experimental parameters, including mobile phase composition (ACN percentage), flow rate (±0.1 mL/min), and column temperature (±5°C). These variations had little effect on retention time, resolution, and peak area, confirming the method's robustness for consistent performance under varying conditions. Additionally, the method showed excellent ruggedness, as it delivered reliable and reproducible results across different operators, instruments, and days, with RSD values consistently below 5%. These findings validated the method's suitability for routine use in diverse laboratory environments, ensuring both precision and reliability.

In summary, the optimized method offers significant improvements in analysis speed, sensitivity, and separation efficiency, making it highly suitable for routine applications in the pharmaceutical and herbal industries. Compared to traditional techniques, which are generally slower, less precise, and less efficient, the proposed method provides clear advantages (Table 6). These results confirm the method's effectiveness for analyzing and quantifying astilbin in *L. strychnifolium* stems, supporting its potential use in quality control and routine analyses. The key advantages of the optimized method are as follows:

1. Reduced analysis time: The method significantly reduces the retention time to 12.10 min and total analysis time to 26 min, achieving a 68.57% reduction compared to traditional methods, which typically take over 60 min.
2. Enhanced sensitivity: With an LOD of 0.10 μg/mL and an LOQ of 0.20 μg/mL, the method offers superior sensitivity compared to other techniques with higher detection thresholds.
3. Improved precision and accuracy: The method demonstrates excellent precision, with intraday and interday RSD values ranging from 0.069% to 1.892%, and recovery rates close to 100%, ensuring accurate quantification of astilbin.
4. Superior separation efficiency: The optimized method provides enhanced separation efficiency, with key chromatographic parameters such as resolution (Rs = 2.141) and theoretical plates (*N* = 10,945), outperforming previously reported methods that show lower values.
5. Robustness and specificity: The method exhibits excellent specificity, with UV spectra of astilbin in extracts matching the standard. The optimized mobile phase and flow rate further improve robustness and reproducibility.
6. Suitability for routine applications: The reduced analysis time and increased efficiency make the method ideal for high-throughput applications, offering reliable results and faster turnaround times, particularly in pharmaceutical and herbal sectors.

These advantages demonstrate that the optimized method is not only time-efficient and practical but also ensures high precision, sensitivity, and reliability, positioning it as an excellent choice for the routine analysis of astilbin in *L. strychnifolium* stems and other matrices.

Finally, we applied the previously demonstrated method to analyze the astilbin content in *L. strychnifolium* stem extracts. The sample of *L. strychnifolium* stem extracts was made by a weight of approximately 5 mg and diluted with 80% ethanol up to 5 mL. Three repetitions of this determination were done in the sample. Stem extracts from *L. strychnifolium* were found to contain 175.51 ± 7.80 μg of astilbin, which was subsequently determined to be 3.51 ± 0.16%.

## 4. Conclusions

This study optimized the process variables of HPLC separation and validation of astilbin in *L. strychnifolium* stems using BBD, a very useful and successful RSM technique. The chromatographic condition that was selected from the factor-level screens was able to significantly reduce the *t*_*R*_ of astilbin. It consisted of several independent factors, such as the percentage of ACN, flow rate, and temperature. The six dependent variables were retention time, peak area, capacity factor, resolution, plate number, and asymmetry factor. The predicted *R*^2^ for the forecast model was similarly significant. All of the models had scores more than 0.7 and a difference between adjusted *R*^2^ and predicted *R*^2^ of less than 0.2, suggesting that they were appropriate and accurate models. It was helpful to analyze the model reduction parameters, outliers, and response transformation. When the astilbin separation temperature was adjusted to 25°C, the percentage of ACN at *t*_0_–*t*_15_ was 19%, and the flow rate was 0.8 mL/min, a significant area in the design space was observed. The astilbin separation in *L. strychnifolium* stem extracts was performed again under optimal HPLC conditions to ensure that the prediction was accurate. The results demonstrated very low percentage errors for every parameter, confirming the accuracy and consistency of the computer software's predictions. A novel approach for developed application as the optimal HPLC condition was generated, resulting in a 26-min shortened working time while maintaining astilbin's *t*_*R*_ of 12.10 min. This resulted in a separation success rate of 68.57% when compared to the original HPLC setting. The sequence of *t*_*R*_, PA, *k*′, Rs, N, and As was found to be 12.108 ± 0.010 min, 78,845,108 ± 420,267, 2.510 ± 0.003, 2.141 ± 0.024, 10,945 ± 80, and 0.991 ± 0.005. All of these findings were within an acceptable range. The limit of detection was 0.10 μg/mL, while the limit of quantitation was 0.20 μg/mL. The calibration curve, which ranged from 0.39 to 50 μg/mL, demonstrated excellent linearity and had an *R*^2^ value of 0.9991. The specificity of the analytical process was underscored by this commonality. The % RSD values for intraday and interday precision ranged from 0.069% to 1.892% and 0.993%–3.229%, respectively. The technique demonstrated precision as shown by recovery values near 100%, notably in the range of 95.56 ± 2.13%–105.57 ± 3.21%. Extracts of *L. strychnifolium*, yielding 12.05 ± 0.14%, were used in the study. An 80% ethanol solution containing 1 mg/mL of the fine extract powder was prepared. The amount of astilbin detected in *L. strychnifolium* stem extracts was 175.51 ± 7.80 μg, which was subsequently determined to be 3.51 ± 0.16%. Therefore, this information may be used to establish criteria for assessing the effectiveness of astilbin as a chemical marker in *L. strychnifolium* stems.

## Figures and Tables

**Table 1:** Experimental design.

	Level		
	Low (−1)	Medium (0)	High (+1)
Factors (independent variables)			
A: percentage of ACN (%)	15	17.5	20
B: flow rate (mL/min)	0.8	1.0	1.2
C: temperature (°C)	25	30	35
Responses (dependent variables)			
*Y*_1_: retention time (min: *t*_*R*_)	Less than 15		
*Y*_2_: peak area (PA)	None		
*Y*_3_: capacity factor (*k*′)	More than 2		
*Y*_4_: resolution (Rs)	More than 2		
*Y*_5_: plate number (N)	More than 2000		
*Y*_6_: asymmetry factor (As)	More than 0.95		

**Table 2:** Gradient systems for screening factor levels by one-factor-at-a-time.

Time (min)	ACN	2% acetic acid
*Gradient System 1 (original condition)*		
0	0	100
5	15	85
25	15	85
40	18	82
45	18	82
50	100	0
60	100	0
61	0	100
70	0	100

*Gradient System 2*		
0	15	85
30	15	85
31	100	0
36	100	0
37	15	85
40	15	85

*Gradient System 3*		
0	17.5	82.5
30	17.5	82.5
31	100	0
36	100	0
37	17.5	82.5
40	17.5	82.5

*Gradient System 4*		
0	20	80
30	20	80
31	100	0
36	100	0
37	20	80
40	20	80

**Table 3:** ANOVA data.

Source	Sum of squares	df	Mean square	*F*-value	*p* value
ANOVA for quadratic model					
Response *Y*_1_: *t*_*R*_					
Model	480.23	9	53.36	467.29	< 0.0001
A—ACN	345.84	1	345.84	3028.72	< 0.0001
B—flow rate	84.70	1	84.70	741.74	< 0.0001
C—temperature	18.73	1	18.73	164.02	< 0.0001
AB	9.51	1	9.51	83.31	< 0.0001
AC	3.77	1	3.77	33.03	0.0007
BC	0.3530	1	0.3530	3.09	0.1221
*A*^2^	15.31	1	15.31	134.10	< 0.0001
*B*^2^	1.31	1	1.31	11.47	0.0116
*C*^2^	0.0200	1	0.0200	0.1750	0.6882
Residual	0.7993	7	0.1142		
Lack of fit	0.7924	3	0.2641	151.91	0.0001
Pure error	0.0070	4	0.0017		
Cor total	481.03	16			
*R* ^2^	0.9983				
Adjusted *R*^2^	0.9962				
Predicted *R*^2^	0.9736				
ANOVA for linear model					
Response *Y*_2_: PA					
Model	5.145E + 15	3	1.715E + 15	125.95	< 0.0001
A—ACN	3.163E + 13	1	3.163E + 13	2.32	0.1515
B—flow rate	5.111E + 15	1	5.111E + 15	375.36	< 0.0001
C—temperature	2.452E + 12	1	2.452E + 12	0.1801	0.6782
Residual	1.770E + 14	13	1.362E + 13		
Lack of fit	1.758E + 14	9	1.954E + 13	65.77	0.0005
Pure error	1.188E + 12	4	2.971E + 11		
Cor total	5.322E + 15	16			
*R* ^2^	0.9967				
Adjusted *R*^2^	0.9591				
Predicted *R*^2^	0.9307				
ANOVA for quadratic model					
Response *Y*_3_: *k*′					
Model	40.35	9	4.48	467.23	< 0.0001
A—ACN	29.06	1	29.06	3028.40	< 0.0001
B—flow rate	7.11	1	7.11	741.45	< 0.0001
C—temperature	1.57	1	1.57	164.10	< 0.0001
AB	0.7993	1	0.7993	83.31	< 0.0001
AC	0.3169	1	0.3169	33.03	0.0007
BC	0.0298	1	0.0298	3.11	0.1214
*A*^2^	1.29	1	1.29	134.13	< 0.0001
*B*^2^	0.1099	1	0.1099	11.45	0.0117
*C*^2^	0.0017	1	0.0017	0.1727	0.6902
Residual	0.0672	7	0.0096		
Lack of fit	0.0666	3	0.0222	151.82	0.0001
Pure error	0.0006	4	0.0001		
Cor total	40.41	16			
*R* ^2^	0.9983				
Adjusted *R*^2^	0.9962				
Predicted *R*^2^	0.9736				
ANOVA for 2FI model					
Response *Y*_4_: Rs					
Model	1.50	6	0.2506	182.76	< 0.0001
A—ACN	0.9969	1	0.9969	727.00	< 0.0001
B—flow rate	0.0996	1	0.0996	72.60	< 0.0001
C—temperature	0.3769	1	0.3769	274.87	< 0.0001
AB	0.0038	1	0.0038	2.80	0.1255
AC	0.0204	1	0.0204	14.91	0.0032
BC	0.0060	1	0.0060	4.37	0.0630
Residual	0.0137	10	0.0014		
Lack of fit	0.0133	6	0.0022	23.33	0.0045
Pure error	0.0004	4	0.0001		
Cor total	1.52	16			
*R* ^2^	0.9910				
Adjusted *R*^2^	0.9855				
Predicted *R*^2^	0.9594				
ANOVA for quadratic model					
Response *Y*_5_: *N*					
Model	6.699E + 06	9	7.443E + 05	115.42	< 0.0001
A—ACN	5356.13	1	5356.13	0.8306	0.3924
B—flow rate	3.059E + 06	1	3.059E + 06	474.33	< 0.0001
C—temperature	2.619E + 06	1	2.619E + 06	406.20	< 0.0001
AB	406.69	1	406.69	0.0631	0.8089
AC	1.815E + 05	1	1.815E + 05	28.14	0.0011
BC	2.426E + 05	1	2.426E + 05	37.61	0.0005
*A*^2^	4.439E + 05	1	4.439E + 05	68.84	< 0.0001
*B*^2^	99,144.85	1	99,144.85	15.38	0.0057
*C*^2^	10,123.23	1	10,123.23	1.57	0.2505
Residual	45,139.06	7	6448.44		
Lack of fit	42,456.53	3	14,152.18	21.10	0.0065
Pure error	2682.53	4	670.63		
Cor total	6.744E + 06	16			
*R* ^2^	0.9933				
Adjusted *R*^2^	0.9847				
Predicted *R*^2^	0.8987				
ANOVA for quadratic model					
Response *Y*_6_: As					
Model	0.0071	9	0.0008	21.64	0.0003
A—ACN	0.0022	1	0.0022	59.83	0.0001
B—flow rate	0.0002	1	0.0002	4.43	0.0733
C—temperature	0.0039	1	0.0039	107.05	< 0.0001
AB	0.0000	1	0.0000	0.5713	0.4744
AC	0.0000	1	0.0000	0.7383	0.4186
BC	0.0002	1	0.0002	4.81	0.0644
*A*^2^	0.0006	1	0.0006	16.38	0.0049
*B*^2^	0.0000	1	0.0000	0.7409	0.4179
*C*^2^	0.0000	1	0.0000	0.7915	0.4032
Residual	0.0003	7	0.0000		
Lack of fit	0.0002	3	0.0001	5.32	0.0702
Pure error	0.0001	4	0.0000		
Cor total	0.0073	16			
*R* ^2^	0.9953				
Adjusted *R*^2^	0.9207				
Predicted *R*^2^	0.5453				

**Table 4:** HPLC chromatographic condition for astilbin separation in *L. strychnifolium* stem extracts.

Time (min)	ACN	2% acetic acid
*Original chromatographic condition (CC)*		
0	0	100
5	15	85
25	15	85
40	18	82
45	18	82
50	100	0
60	100	0
61	0	100
70	0	100

*Optimal chromatographic condition (OCC)*		
0	19	81
30	19	81
31	100	0
36	100	0
37	19	81
40	19	81

*Chromatographic condition for validation method (CCV)*		
0	19	81
15	19	81
16	100	0
21	100	0
22	19	81
26	19	81

**Table 5:** Verification of model predictions with experimental results and percentage error estimations.

Responses	Predicted value	Actual value (*n* = 3)	Error (%)
*t*_*R*_ (min)	11.94	12.04 ± 0.01	0.83
PA	126,861,789.35	124,637,241 ± 402,748	−1.78
*k*′	2.46	2.49 ± 0.00	1.21
Rs	2.59	2.53 ± 0.02	−2.35
*N*	8840.79	8621 ± 115	−2.55
As	0.96	0.93 ± 0.00	−3.50

**Table 6:** Summary the comparison of optimized HPLC method with previously reported methods for astilbin analysis.

Parameters	Current study (optimized method)	Other studies [13, 22, 45–47]
Retention time (*t*_*R*_)	12.10 min	> 31.50 min (some studies report > 60 min)

Mobile phase	Gradient: 19% ACN + 2% acetic acid	Gradient or isocratic systems, varying ACN/water ratios (e.g., 20%–80%)

Flow rate	0.8 mL/min	1.0–1.2 mL/min

Column temperature	25°C	30°C–40°C in some studies

Limit of detection (LOD)	0.10 μg/mL	Mostly > 0.20 μg/mL (not always reported in the literature)

Limit of quantitation (LOQ)	0.20 μg/mL	Mostly > 0.50 μg/mL

Analysis time	26 min (∼68.57% reduction)	> 70 min (in original systems)

Precision	Intraday RSD: 0.069% to 1.892% Interday precision RSD: 0.993%–3.229%	Intraday RSD: 2.0–3.5% Interday precision RSD: 2.0%–4.5% (may be higher in some cases)

Linear range	0.39–50 μg/mL (*R*^2^ value of 0.9991)	1.0–100 μg/mL (comparable but with lower regression, *R*^2^ < 0.9991 in many cases)

Specificity	High specificity (UV spectra match standard astilbin)	Some studies report interference from herbal extract peaks

Separation efficiency	Rs = 2.141, *N* = 10,945	Rs = 1.5–2.0, *N* < 5000 in several studies

## Data Availability

The data that support the findings of this study are available from the corresponding author upon reasonable request.

## References

[1] Panche A. N., Diwan A. D., Chandra S. R. (2016). Flavonoids: An Overview. *Journal of Nutrition Sciences*.

[2] Thepphakhun T., Intanon S. (2020). Total Phenolics, Flavonoids, Antioxidant Activity, and Allelopathic Potential of Praxelis. *J Curr Sci Technol*.

[3] Maleki S. J., Crespo J. F., Cabanillas B. (2019). Anti-Inflammatory Effects of Flavonoids. *Food Chemistry*.

[4] Kopustinskiene D. M., Jakstas V., Savickas A., Bernatoniene J. (2020). Flavonoids as Anticancer Agents. *Nutrients*.

[5] Shen N., Wang T., Gan Q., Liu S., Wang L., Jin B. (2022). Plant Flavonoids: Classification, Distribution, Biosynthesis, and Antioxidant Activity. *Food Chemistry*.

[6] Tungmunnithum D., Thongboonyou A., Pholboon A., Yangsabai A. (2018). Flavonoids and Other Phenolic Compounds from Medicinal Plants for Pharmaceutical and Medical Aspects: An Overview. *Medicine (Baltimore)*.

[7] Dias M. C., Pinto D. C. G. A., Silva A. M. S. (2021). Plant Flavonoids: Chemical Characteristics and Biological Activity. *Molecules*.

[8] Mutha R. E., Tatiya A. U., Surana S. J. (2021). Flavonoids as Natural Phenolic Compounds and Their Role in Therapeutics: An Overview. *Futur J Pharm Sci*.

[9] Nakahara T., Nishitani Y., Nishiumi S., Yoshida M., Azuma T. (2017). Astilbin From *Engelhardtia Chrysolepis* Enhances Intestinal Barrier Functions in Caco-2 Cell Monolayers. *European Journal of Pharmacology*.

[10] Kedrina-Okutan O., Novello V., Hoffmann T. (2018). Constitutive Polyphenols in Blades and Veins of Grapevine (*Vitis vinifera* L.) Healthy Leaves. *Journal of Agricultural and Food Chemistry*.

[11] Pérez-Nájera V. C., Gutiérrez-Uribe J. A., Antunes-Ricardo M. (2018). *Smilax aristolochiifolia* Root Extract and its Compounds Chlorogenic Acid and Astilbin Inhibit the Activity of *α*-Amylase and *α*-Glucosidase Enzymes. *Evidence-based Complementary and Alternative Medicine: eCAM*.

[12] Huang L., Deng J., Chen G. (2019). The Anti-Hyperuricemic Effect of Four Astilbin Stereoisomers in *Smilax glabra* on Hyperuricemic Mice. *Journal of Ethnopharmacology*.

[13] Sampaopan Y., Suksaeree J. (2022). Formulation Development and Pharmaceutical Evaluation of *Lysiphyllum Strychnifolium* Topical Patches for Their Anti-Inflammatory Potential. *AAPS PharmSciTech*.

[14] Zhang Q.-F., Nie H.-C., Shangguang X.-C., Yin Z.-P., Zheng G.-D., Chen J.-G. (2013). Aqueous Solubility and Stability Enhancement of Astilbin Through Complexation With Cyclodextrins. *Journal of Agricultural and Food Chemistry*.

[15] Guo J., Qian F., Li J., Xu Q., Chen T. (2007). Identification of a New Metabolite of Astilbin, 3′-O-Methylastilbin, and its Immunosuppressive Activity against Contact Dermatitis. *Clinical Chemistry*.

[16] Zhang C.-y., Zhu J.-y., Ye Y. (2017). Erhuang Formula Ameliorates Renal Damage in Adenine–Induced Chronic Renal Failure Rats via Inhibiting Inflammatory and Fibrotic Responses. *Biomedicine & Pharmacotherapy*.

[17] Tangnak N., Hongtrakul V., Keeratinijakal V. (2018). Analysis of Genetic Diversity Evaluation of *Lysiphyllum Strychnifolium* (Craib) A. Schmitz in Thailand Using Amplified Fragment Length Polymorphism Markers. *Agriculture and Natural Resources*.

[18] Itharat A., Sayompark S., Hansakul P., Dechayont B. (2016). In Vitro Antioxidant Activities of Extracts of *Bauhinia Strychnifolia* Stems and Leaves: Comparison With Activities in Green Tea Extracts. *Medicinal & Aromatic Plants*.

[19] Maitree M., Hirunpanich Sato V., Sithisarn P., Chewchinda S. (2018). Evaluation of Antioxidant Activities and HPTLC Analysis of *Lysiphyllum Strychnifolium* (Craib) A. Schmitz Leaf Extract. *Thai Journal of Pharmaceutical Sciences*.

[20] Sutiyaporn A., Hirunpanich Sato V., Parichatikanond W., Chewchinda S. (2018). The Study of Antihyperuricemic Effect of *Lysiphyllum Strychnifolium* (Craib) A. Schmitz Leaf Extract. *Thai Journal of Pharmaceutical Sciences*.

[21] Kongkiatpaiboon S., Duangdee N., Tayana N., Schinnerl J., Bacher M., Chewchinda S. (2020). Yanangdaengin, a Dihydrochalcone Glucoside Galloyl Ester as Active Antioxidative Agent From Leaves of *Lysiphyllum Strychnifolium* (Syn. *Bauhinia Strychnifolia*). *Chinese Herbal Medicines*.

[22] Pichayakorn W., Monton C., Sampaopan Y., Panrat K., Suksaeree J. (2022). Fabrication and Characterization of Buccal Film Loaded Self-Emulsifying Drug Delivery System Containing *Lysiphyllum Strychnifolium* Stem Extracts. *AAPS PharmSciTech*.

[23] Mourabet M., El Rhilassi A., El Boujaady H., Bennani-Ziatni M., Taitai A. (2017). Use of Response Surface Methodology for Optimization of Fluoride Adsorption in an Aqueous Solution by Brushite. *Arabian Journal of Chemistry*.

[24] Yolmeh M., Jafari S. M. (2017). Applications of Response Surface Methodology in the Food Industry Processes. *Food and Bioprocess Technology*.

[25] Zaid H., Al-Sharify Z. T., Hamzah M. H., Rushdi S. (2022). Optimization of Different Chemical Processes Using Response Surface Methodology-A Review. *Journal of Engineering and Sustainable Development*.

[26] Salagarkar S., Salunkhe S., Doke C. (2023). A Review on Box-Behnken Design for Analytical Method Development. *International Journal of Modern Research in Engineering and Technology*.

[27] Czyrski A., Sznura J. (2019). The Application of Box-Behnken-Design in the Optimization of HPLC Separation of Fluoroquinolones. *Scientific Reports*.

[28] Elkady E. F., Fouad M. A., Mozayad A. N. (2022). Application of Box-Behnken Experimental Design and Response Surface Methodology for Selecting the Optimum RP-HPLC Conditions for the Simultaneous Determination of Methocarbamol, Indomethacin and Betamethasone in Their Pharmaceutical Dosage Form. *BMC Chemistry*.

[29] Haque S. M., Rahman H., Rahman N. (2023). Application of Box–Behnken Design Combined Response Surface Methodology to Optimize HPLC and Spectrophotometric Techniques for Quantifying Febuxostat in Pharmaceutical Formulations and Spiked Wastewater Samples. *Microchemical Journal*.

[30] Hnin H. M., Tun T., Jansook P. (2024). Development and Validation of High-Performance Liquid Chromatography Method for the Simultaneous Quantification of Rivastigmine Hydrogen Tartrate and Asiaticoside Co-Loaded in Niosomes: A Box–Behnken Design Approach. *Journal of Chromatography B*.

[31] Elkady E., Fouad M., Mozayad A. (2023). Application of Box–Behnken Design and Response Surface Methodology for Selecting the Optimum RP-HPLC Conditions for the Simultaneous Determination of Paracetamol and Diclofenac Sodium along with Three Skeletal Muscle Relaxants in Three Different Pharmaceutical Dosage Forms. *Journal of Chromatographic Science*.

[32] Haque S. M. (2022). Box–Behnken Experimental Design for Optimizing the HPLC Method to Determine Hydrochlorothiazide in Pharmaceutical Formulations and Biological Fluid. *Journal of Molecular Liquids*.

[33] Khorshidi N., Rahimi M., Salimikia I. (2020). Application of Aeration-Assisted Homogeneous Liquid–Liquid Microextraction Procedure Using Box–Behnken Design for Determination of Curcumin by HPLC. *Journal of Separation Science*.

[34] Kumar G., Mullick P., Nandakumar K., Mutalik S., Rao C. M. (2022). Box–Behnken Design-Based Development and Validation of a Reverse-Phase HPLC Analytical Method for the Estimation of Paclitaxel in Cationic Liposomes. *Chromatographia*.

[35] Alam P., Shakeel F., Taleuzzaman M. (2022). Box-Behnken Design (BBD) Application for Optimization of Chromatographic Conditions in RP-HPLC Method Development for the Estimation of Thymoquinone in *Nigella sativa* Seed Powder. *Processes*.

[36] Ferreira N., Viana T., Henriques B. (2023). Application of Response Surface Methodology and Box–Behnken Design for the Optimization of Mercury Removal by *Ulva* Sp. *Journal of Hazardous Materials*.

[37] Bose A. (2014). HPLC Calibration Process Parameters in Terms of System Suitability Test. *Austin Chromatography*.

[38] Singh V., Haque S., Niwas R., Srivastava A., Pasupuleti M., Tripathi C. K. M. (2016). Strategies for Fermentation Medium Optimization: An In-Depth Review. *Frontiers in Microbiology*.

[39] Abdel-Rahman M. A., Hassan S. E. D., El-Din M. N. (2020). One-Factor-at-a-Time and Response Surface Statistical Designs for Improved Lactic Acid Production From Beet Molasses by *Enterococcus Hirae* Ds10. *SN Applied Sciences*.

[40] Bhaturiwala R., Bagban M., Mansuri A., Modi H. (2022). Successive Approach of Medium Optimization Using One-Factor-at-a-Time and Response Surface Methodology for Improved *β*-Mannanase Production from *Streptomyces* Sp. *Bioresource Technology Reports*.

[41] Zhang D. (2017). A Coefficient of Determination for Generalized Linear Models. *The American Statistician*.

[42] Minitab L. (2013). Multiple Regression Analysis: Use Adjusted R-Squared and Predicted R-Squared to Include the Correct Number of Variables. https://blog.minitab.com/en/adventures-in-statistics-2/multiple-regession-analysis-use-adjusted-r-squared-and-predicted-r-squared-to-include-the-correct-number-of-variables.

[43] Sureiman O., Mangera C. M. (2020). F-Test of Overall Significance in Regression Analysis Simplified. *Journal of the Practice of Cardiovascular Sciences*.

[44] Costa S., Barroso M., Castañera A., Dias M. (2010). Design of Experiments, a Powerful Tool for Method Development in Forensic Toxicology: Application to the Optimization of Urinary Morphine 3-glucuronide Acid Hydrolysis. *Analytical and Bioanalytical Chemistry*.

[45] Du Q., Li L., Jerz G. (2005). Purification of Astilbin and Isoastilbin in the Extract of *Smilax glabra* Rhizome by High-Speed Counter-Current Chromatography. *Journal of Chromatography A*.

[46] Zhang Q.-F., Fu Y.-J., Huang Z.-W., Shangguang X.-C., Guo Y.-X. (2013). Aqueous Stability of Astilbin: Effects of pH, Temperature, and Solvent. *Journal of Agricultural and Food Chemistry*.

[47] Sampaopan Y., Kitprapiumpon N., Kongkiatpaiboon S., Duangdee N., Wongyai S. (2021). Isolation and HPLC Analysis of Astilbin in *Lysiphyllum strychnifolium* (Syn. *Bauhinia strychnifolia*) Stem. *Science & Technology Asia*.

